# Nanomedicine Strategies for Heating “Cold” Ovarian Cancer (OC): Next Evolution in Immunotherapy of OC

**DOI:** 10.1002/advs.202202797

**Published:** 2022-07-22

**Authors:** Yuqi Yang, Tianjiao Zhao, Qiaohui Chen, Yumei Li, Zuoxiu Xiao, Yuting Xiang, Boyu Wang, Yige Qiu, Shiqi Tu, Yitian Jiang, Yayun Nan, Qiong Huang, Kelong Ai

**Affiliations:** ^1^ Department of Pharmacy Xiangya Hospital Central South University Changsha Hunan 410008 P. R. China; ^2^ National Clinical Research Center for Geriatric Disorders Xiangya Hospital Central South University Changsha Hunan 410008 P. R. China; ^3^ Xiangya School of Pharmaceutical Sciences Central South University Changsha Hunan 410078 P. R. China; ^4^ Hunan Provincial Key Laboratory of Cardiovascular Research Xiangya School of Pharmaceutical Sciences Central South University Changsha Hunan 410078 P. R. China; ^5^ Department of Assisted Reproduction Xiangya Hospital Central South University Changsha Hunan 410008 P. R. China; ^6^ Geriatric Medical Center People's Hospital of Ningxia Hui Autonomous Region Yinchuan Ningxia 750002 P. R. China

**Keywords:** immunotherapy, nanomaterials, nanomedicines, ovarian cancer, tumor immune microenvironment

## Abstract

Immunotherapy has revolutionized cancer treatment, dramatically improving survival rates of melanoma and lung cancer patients. Nevertheless, immunotherapy is almost ineffective against ovarian cancer (OC) due to its cold tumor immune microenvironment (TIM). Many traditional medications aimed at remodeling TIM are often associated with severe systemic toxicity, require frequent dosing, and show only modest clinical efficacy. In recent years, emerging nanomedicines have demonstrated extraordinary immunotherapeutic effects for OC by reversing the TIM because the physical and biochemical features of nanomedicines can all be harnessed to obtain optimal and expected tissue distribution and cellular uptake. However, nanomedicines are far from being widely explored in the field of OC immunotherapy due to the lack of appreciation for the professional barriers of nanomedicine and pathology, limiting the horizons of biomedical researchers and materials scientists. Herein, a typical cold tumor‐OC is adopted as a paradigm to introduce the classification of TIM, the TIM characteristics of OC, and the advantages of nanomedicines for immunotherapy. Subsequently, current nanomedicines are comprehensively summarized through five general strategies to substantially enhance the efficacy of immunotherapy by heating the cold OC. Finally, the challenges and perspectives of this expanding field for improved development of clinical applications are also discussed.

## Graphical abstract

Biological characteristics and immunosuppressive mechanisms of the cold tumor immune microenvironment (TIM) are described. Nanomedicines modulating TIM and improving the antitumor responses via various mechanisms are presented. Strategies and progress of nanomedicines for immunotherapy are systematically reviewed. Urgent challenges and potential future directions of immune‐nanomedicines are discussed.

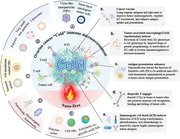

## 1 Introduction

**Figure 1:**
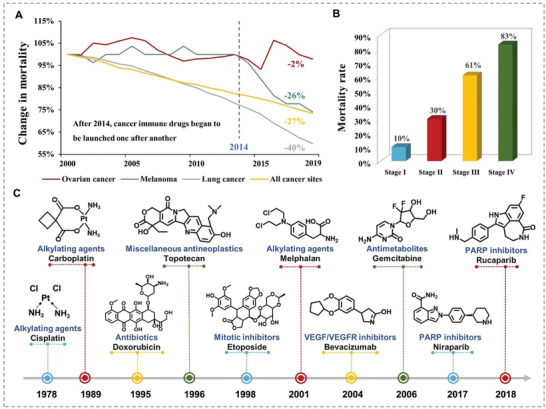
Current status of OC treatment. A) Changes in mortality of OC, melanoma, lung cancer, and all sites. B) Mortality of different stages of OC. C) The evolution timeline of OC systemic therapy.

The overall cancer mortality rate decreased by 32% from 1991 to 2019 with the continuous exploration of new treatments.^[^ ^1^ ^]^ Particularly, emerging immunotherapy has led to a significant decline in cancer mortality, with an 8.2% drop in just five years from 2015 to 2019.^[^ ^2^ ^]^ For instance, the melanoma death rate has decreased by an average of 5.7% annually after the US Food and Drug Administration (FDA) approved Bristol Myers Squibb's PD‐1 monoclonal antibody ipilimumab for the treatment of advanced melanoma in 2014.^[^ ^1^, ^3^, ^4^ ^]^ Currently, immunotherapy has also been approved for the treatment of nonsmall cell lung cancer, nasopharyngeal cancer, and advanced cutaneous squamous cell carcinoma, for which immunotherapy has shown amazing therapeutic effects with a significantly declined mortality^[^ ^5^ ^]^ (**Figure** **1**A).

Unfortunately, immunotherapy still does not benefit ovarian cancer (OC) patients.^[^ ^6^ ^]^ Currently, only one immunotherapy‐dostarlimab has been approved by FDA for OC treatment.^[^ ^7^, ^8^ ^]^ OC is one of the most dangerous gynecological tumors. In 2020, the number of new cases and deaths from OC was as high as 314 000 and 207 000 in the world, respectively.^[^ ^9^ ^]^ OC already accounts for 5% of female cancer‐related deaths and has become the fifth leading cause of female cancer‐related deaths.^[^ ^1^ ^]^ OC has few symptoms in the early stage because the ovary hangs in the abdominal cavity and has a less abundant nerve ending distribution.^[^ ^10^ ^]^ Most OCs are diagnosed in the middle and late stages, at which time OC is usually already widely metastasized to many important organs through ascites or peritoneum.^[^ ^11^ ^]^ Therefore, OC is also considered the “silent killer”^[^ ^12^ ^]^ (Figure 1B). Many therapeutic methods and drugs have been developed for OC treatment, such as cyclophosphamide, doxorubicin, paclitaxel, cisplatin, etc.^[^ ^13^, ^14^ ^]^ For example, a standard treatment for advanced epithelial OC (the most common type of OC, accounting for 90%) is the surgical removal of the bulk tumor, followed by chemotherapy with paclitaxel and carboplatin.^[^ ^15^ ^]^ In recent years, molecular targeted drugs, such as angiogenesis inhibitors, DNA repair inhibitors, etc., have also been approved by the FDA for OC treatment^[^ ^16^, ^17^ ^]^ (Figure 1C). However, these treatments have had limited success, showing up to 70% of epithelial OC recurrence rates after treatment.^[^ ^18^ ^]^

The prognosis of OC is closely related to tumor immunity.^[^ ^19^ ^]^ OC cells also express many cancer‐related antigens, such as epithelial cell adhesion molecule, cancer antigen 125, *α*‐folate receptor, etc.^[^ ^20^, ^21^, ^22^ ^]^ These indicate that immunotherapy is very promising in OC treatment.^[^ ^23^ ^]^ Currently, many clinical trials on OC are being performed to facilitate the clinical translation of immunotherapy.^[^ ^24^, ^25^, ^26^ ^]^ As of May 09, 2022, FDA has approved a total of 183 clinical trials involving OC immunotherapy, of which 43 and 29 have been completed and terminated (discontinued due to determination of ineffectiveness), respectively, and most experiments have not been optimistic in these completed clinical trials.^[^ ^27^ ^]^ For example, only 10–15% of OC are positive for immune checkpoint inhibitors (ICI).^[^ ^28^ ^]^ The main reason for this dilemma is that OC has a “cold” tumor immune microenvironment (TIM) relative to lung cancer and melanoma, where immunosuppressive cells are abundant, and tumor‐specific immune cells are rarely present.^[^ ^29^ ^]^ Immune drugs, such as ICIs, have a disappointing therapeutic effect on OC because these drugs need sufficient immune cells in the TIM to act.^[^ ^30^ ^]^

**Figure 2:**
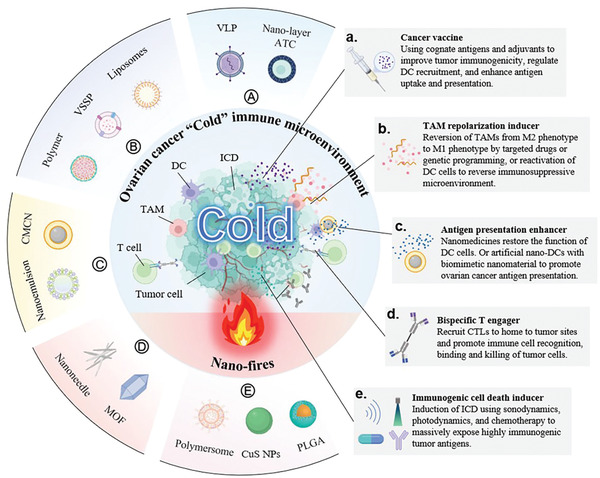
Schematic illustration of developing nanomaterials in OC immunotherapy. A–E) Nanomaterials used in exploring new treatments for cancer vaccines: TAM repolarization inducer, antigen presentation enhancer, BiTEs, and ICD inducers. (a–e) Therapeutic effects and mechanisms of cancer vaccines: TAM repolarization inducer, antigen presentation enhancer, BiTEs, and ICD inducers in the TIM of OC.

Recently, substantial progress in nanomedicines has shed new light on OC immunotherapy.^[^ ^31^ ^]^ Nanomedicines have many advantages in cancer immunotherapy, such as greatly improving the bioavailability of immune drugs, reducing side effects, and promoting the enrichment of drugs at tumor sites, abdominal cavity, or lymph nodes^[^ ^32^, ^33^, ^34^ ^]^ (**Figure** **2**A–E). Some emerging tailored nanomedicines have demonstrated potent immunotherapeutic effects on OC by exposing the internal antigens of OC cells, increasing the infiltration of antigen‐presenting cells (APCs) and T lymphocytes, and reversing the TIM (Figure 2a–e).^[^ ^35^, ^36^ ^]^ Despite the various dazzling advantages, nanomedicines for OC immunotherapy are far from being fully explored and largely underappreciated by nanomedicine researchers. The lack of appreciation for the potential of nanomedicines in OC immunotherapy can be attributed to professional barriers limiting the horizons of OC experts and materials scientists. Limited understanding of the unique pathological properties of OC by most materials researchers causes research to be unable to directly address the pain points of OC treatment. Additionally, most biomedical researchers lack sufficient understanding of the abundant properties of nanomedicines to rationally apply nanomaterials in the OC immunotherapies. For the first time, this review highlights and comments on recent advances in nanotechnology for OC immunotherapy. We briefly discuss the TIM of OC to provide a solid background of OC pathology and then focus on the most significant advances and detailed discussions of nanomedicines for immunotherapy. Ultimately, we also provide a thorough discussion on the prospects and future challenges of nanomedicines in these emerging research frontiers. We believe that this review will provide new insights into the application of nanomedicines in OC immunotherapy and further facilitate the development of this research field.

## 2 OC TIM and Immunotherapy

**Figure 3:**
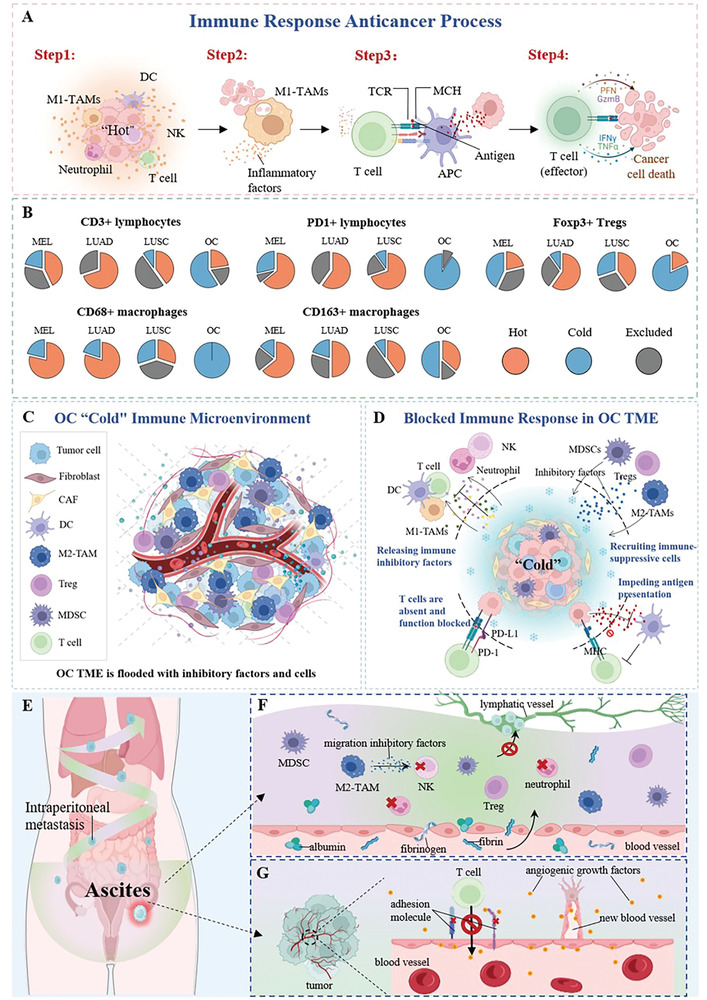
The immune process and how it is hindered by the cold TIM of OC. A) Four steps of an anticancer immune response. Step 1: tumor site is infiltrated with various immunocompetent cells and factors. Step 2: macrophages engulf necrotic tumor cells and release inflammatory cytokines. Step 3: DCs capture tumor cells and present antigens to T cells. Step 4: effector T cells kill tumor cells by releasing toxic substances to tumor cells. B) Analysis of all five immune cell types and four analyzed tumor types (MEL = melanoma, LUAD = lung adenocarcinoma, LUSC = lung squamous cancer, OC = ovarian cancer). These data comprise all 965 tissue slides from 177 patients. C) OC “cold” immune microenvironment. D) Pathways of OC TIM to block anticancer immunity response. E–G) A biological barrier preventing immune cell infiltration and migration in OC. (E) OC cells disseminate throughout the peritoneal cavity and metastasize across body cavities through ascites. (F) Permeability of peritoneal capillaries and blockage of lymphatic return by cancer cells lead to ascites. Ascites recruits immunosuppressive cells, suppresses neutrophils, and prompts TAMs to release migration inhibitory factors to stop the tumor‐killing ability of natural killer (NK) cells. (G) The release of angiogenic growth factors in OC inhibits the expression of adhesion molecules, preventing T cells from adhering to and traversing the endothelium.

Cancer can trigger an immune response, and many classes of inflammatory factors and immune cells are widely distributed in various tumor tissues.^[^ ^37^ ^]^ Cancer immunity involves both innate immune and adaptive immune systems, and the typical process is as follows. APCs, including macrophages, B cells, and dendritic cells (DCs), especially DCs, present cancer antigens to naive T cells with major histocompatibility complex (MHC) I/II molecules to initiate a specific immune response by phagocytosis (e.g., macrophages and DCs) or receptor‐mediated endocytosis (B cells). Naive T cells are activated to differentiate into cytotoxic T cells (CTLs). Finally, CTLs directly lyse tumor cells and indirectly kill tumor cells by secreting cytokines, such as gamma interferon (IFN‐*γ*)^[^ ^38^ ^]^ (**Figure** **3**A). However, solid tumors can alter the immune cell phenotype and reshape the TIM, such as the expression of immune checkpoint‐related proteins, to suppress the activity of effector T cells and induce the inflammatory M1 tumor‐associated macrophages (TAMs) phenotype to transform into the anti‐inflammatory M2 type. Therefore, the efficacy of immunotherapy is closely related to the inflammatory state at the tumor site.^[^ ^39^ ^]^

According to Daniel's definition, TIMs are divided into three types: hot tumor, excluded tumor, and cold tumor.^[^ ^40^ ^]^ Hot tumors are characterized by many immune cells infiltrating the solid tumor from the periphery to the center. Excluded tumors are those in which immune cells only accumulate at the periphery of solid tumors. Cold tumors, also known as immune deserts, are characterized by little or no immune cell infiltration. Recently, Jakob has examined nearly a thousand pathological sections of tumor tissue from cancer patients and stratified different types of cancer according to the distribution of their immune cells.^[^ ^41^ ^]^ As shown in Figure 3B–D and **Table** **1**, OC contains few inflammatory cells^[^ ^29^ ^]^ and represents a typical cold tumor. For example, OC hardly contains CD68+ macrophages (M1) and PD1+ lymphocytes and only comprises a small number of CD3+ CD8+ cell lymphocytes. Conversely, OC contains abundant anti‐inflammatory CD163+ macrophages (M2).^[^ ^42^ ^]^

**Table 1:** Immune cells in OC microenvironment

Cell	Activated by	Expressed proteins	Function	Refs.
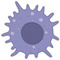 Myeloid‐derived suppressor cell (MDSC)	GM‐CSF, CXCL1, VEGF, CXCL2, IL‐1, PGE2, CXCL5, MIF	IL‐10, IL‐23, TGF‐*β*, Arginase 1, CCR2, CD38, CD80, CD124	Immunosuppressive cell. Inhibits T cell proliferation and homing. Promotes the transformation of M2 type. Blocks NK killing ability.	[46, 47]
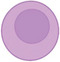 Treg	CCL5, PGE2, IL‐2, TGF‐*β*, galectin 1	TGF‐*β*, IL‐10, IL‐35, *β*‐GBP, CD4, CD25, FoxP3	Immunosuppressive cell. Mediates immune tolerance. Blocks normal recognition and destruction of the immune system.	[48, 49, 50]
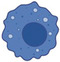 M2‐TAM	CCL2, CSF1, CXCL12, TNF, VEGF‐A, IL‐4, SEMA3A, CCL22	PD‐L1, IL‐10, B7‐H4, TGF‐*β*, VEGF, arginase1, CD163, CD206, CD209	Immunosuppressive cell. Regulates T cell suppressive response. Promotes tumor angiogenesis and metastasis.	[51, 52, 53]
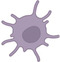 Regulatory DC (DCreg)	VEGF, TGF‐*β*	IL‐10, NO, TGF‐*β*, IDO, MHC, ILT3/4	Immunosuppressive cell. Induces Treg production. Inhibits T cell function.	[54, 55]
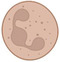 N2‐Tumor‐associated neutrophil (TAN)	CXCL15, HMGB1, TGF‐*β*	VEGF, MMP9, IL‐8, CCL2, CCL17, CD16, CD11b	Immunosuppressive cell. Promotes tumor metastasis. Recruits Treg.	[56, 57]
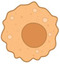 M1‐TAM	IFN‐*γ*, LPS, CSF2	IL‐1*β*, IL‐12p40, IL‐6, MIP1‐*α*, TNF‐*α*, CXCL9, CD68, CD80, iNOS	The first line of defense of immunity. Exerts antigen‐presenting ability. Induces a strong Th1 response.	[58]
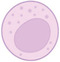 NK cell	IL‐18, IL‐12, IL‐27, IL‐15, IFN‐*γ*	IFN‐*γ*, Granzyme, TNFs, Perforin, CD16, NKp30, NKG2D, FasL, DNAM‐1	The first line of defense for immunity. Kills tumor cells directly or indirectly without antigen stimulation.	[59, 60]
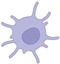 DC	GM‐CSF, LPS, TSLP, IL‐4, IFN‐*γ*	TNF‐*α*, INF‐*α*, IL‐1, IL‐12, IL‐15, IL‐18, MHC, CD86, CD40	The strongest antigen presenting cell. Activates cytotoxic T lymphocytes and NK cells.	[61]
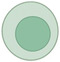 CTL	IL‐2, IL‐4, IL‐18, IL‐12, IL‐15	IFN‐*γ*, IL‐2, Perforin, Granzyme, TNF‐*β*, CD3, CD4, CD8, TCR, FasL	Specifically kills antigen‐bearing tumor cells. Promotes cytolysis and induces tumor cell apoptosis.	[62, 63]
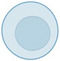 T helper cell 17 (Th17)	IL‐6, TGF‐*β*, IL‐23	IL‐17A/F, IL‐22, CCL20, CD161, CD49b, CCR6	Recruits neutrophils. Mediates inflammatory responses.	[64, 65]
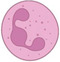 N1‐TAN	SM16, IFN‐*β*	IL‐18, CXCR1/2, CCL3, CXCL10, TNF*α*, NETs, G‐CSFR	Presents antigens to activate T cells. Recruits and activate NK cells.	[57, 66]

Additionally, OC also has a biological barrier to prevent immune cell infiltration and migration. OC hanging in the abdominal cavity can secrete many classes of growth factors and immunosuppressive factors into the ascites to induce peritoneal angiogenesis and increase the permeability of capillaries.^[^ ^43^ ^]^ In this case, OC cells accelerate their migration into the peritoneum and vital internal organs (Figure 3E), subsequently blocking the lymphatic vessels to prevent inflammatory cell migration^[^ ^44^ ^]^ (Figure 3F). Furthermore, abundant immunosuppressive factors in ascites from TIM can further prevent the infiltration and migration of immune cells^[^ ^45^ ^]^ (Figure 3G).

## 3 Advantages of Nanomedicine for “Heating” OC

**Figure 4:**
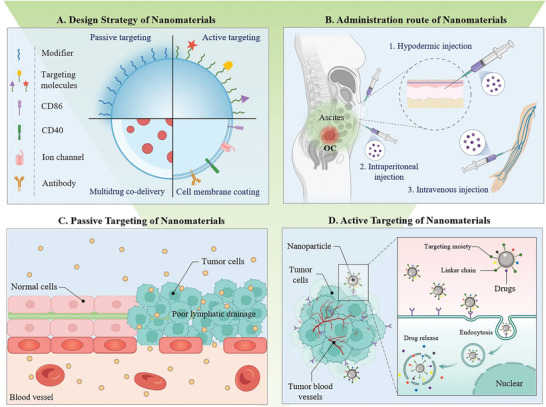
Development advantages of nanomaterials in OC immunotherapy. A) Four drug development strategies owing to the superior properties of nanomaterials. B) Three commonly used administration routes of nanomedicines in OC treatment. C) Nanomedicines achieve passive targeting through the EPR effect. They enter the tumor target through irregular intercellular spaces at the cancerous site. D) Nanomedicine surface modification with specific targeting molecules to achieve active targeting by recognizing and binding to tumor cell surface targets.

Fortunately, tailored nanomedicines can reshape the “cold” immune state of OC and greatly improve the antitumor activity of immunotherapy. Nanomedicines have many natural advantages in OC immunotherapy (**Figure** **4**A). First, many APCs, such as TAMs and DCs, naturally have the property of phagocytizing nanoparticles to greatly improve the targeting of nanomedicines for immunotherapy. For example, Haber et al. have found that TAMs can specifically phagocytize negatively charged nanoparticles larger than 100 nm. Taking 200 nm silica nanoparticles as an example, 80% of the nanoparticles injected intratumorally have remained in OC lesions, of which 87% were phagocytized by TAMs.^[^ ^67^ ^]^ Second, the natural biological barrier of OC provides a great opportunity for nanomedicines to remain in OC lesions, ascites, and OC intraperitoneal metastases. For example, nanomedicines can reside in the abdominal cavity for a long time because the larger particles are difficultly excreted into the blood circulation after intraperitoneal injection, while small‐molecule drugs are easily excreted from ascites into the blood circulation by passive diffusion^[^ ^68^, ^69^ ^]^ (Figure 4B). Additionally, the enhanced permeability and retention (EPR) effect increases the efflux of traditional small‐molecule drugs from OC sites but enables nanomedicines to be effectively confined to OC lesions^[^ ^70^, ^71^ ^]^ (Figure 4C). Third, the controlled synthesis of nanomaterials provides a solid foundation for tailored nanomedicines.^[^ ^72^ ^]^ In theory, the desired nanomedicines with high targeting and efficiency can be prepared by controlling a series of physicochemical parameters, such as size, morphology, surface modification, and composition, for immunotherapy according to the pathological characteristics of OC.^[^ ^73^, ^74^, ^75^ ^]^ For example, there are many specific and highly expressed antibodies on the cell surface in the TIM of OC, and the targeting of nanomedicines can be greatly improved by specifically modifying the corresponding ligands^[^ ^76^ ^]^ (Figure 4D).

Based on those significant advantages, these emerging nanomedicines have demonstrated great success in improving biodistribution (e.g., circulation time and tumor tissue homing and penetration), sustainable properties (e.g., through sustained release and improved immune memory), and efficacy (e.g., through adjuvant materials and therapeutic synergy) of immune drugs for OC. According to the immune mechanism of nanomedicines, these therapies focus on the following five aspects. 1) Nanovaccines activate “cold” TIM by triggering innate immunity in OC. 2) Nanomedicines repolarize M2‐type TAMs to immunocompetent M1‐type in OC. 3) Nanomedicines activate specific immune responses by enhancing the efficacy of antigen presentation to T cells. 4) Nanomedicines recruit and promote CTLs to kill OC cells by bispecific T engagers (BiTEs). 5) Nanomedicines as a multifunctional platform induce immunogenic cellular death (ICD) to “heat” OC by combining chemotherapy and phototherapy.

## 4 Nanovaccines “Heating” OC

**Figure 5:**
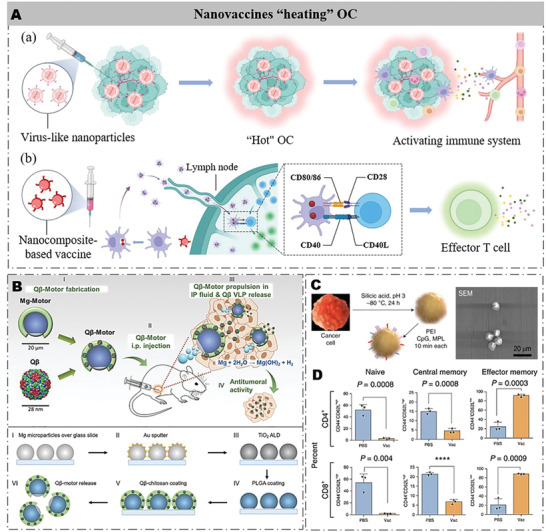
Strategies of nanovaccine in OC immunotherapy. A) Schematic illustration of the therapeutic mechanism of nanovaccines in OC. (a) Adjuvanted cancer vaccines are injected into OC tumor sites to induce an immune response by modulating DC recruitment, enhancing antigen presentation, and priming naive T cells. (b) Adjuvant and antigen composite cancer vaccines enter the lymph to actively deliver and present OC antigens and activate naive T cells. B) Schematic (not to real scale) of the fabrication, in vivo administration, and in vivo actuation of Q*β*‐motors as an active Q*β* VLP release alternative in ovarian tumors. Additional schematic (not to real scale) of the Q*β*‐motors preparation. Reproduced with permission.^[^ ^85^ ^]^ Copyright 2020, Wiley‐VCH. C) Cryosilicification and adsorption of PAMPs to cancer cells. Blue motifs, CpG; purple motifs, MPL; red motifs, PEI. D) Percent and number of IP CD4+ and CD8+ T cells with naive (CD44− CD62L high), central memory (CD44+ CD62L high), and effector memory (CD44+ CD62L low) phenotypes. Reproduced with permission.^[^ ^89^ ^]^ Copyright 2019, Springer Nature.

Tumor vaccines, as a form of active immunotherapy, have attracted much attention due to their high safety and minimal side effects.^[^ ^77^ ^]^ However, OC vaccines have a poor immune response due to the low immunogenicity and instability of OC antigens.^[^ ^48^ ^]^ Nanovaccines can greatly improve the effect of OC immunotherapy by the following methods. First, highly immunogenic nanovaccines administered to the OC site act as an immune adjuvant to change the TIM of OC from cold to hot and induce adaptive immunity to kill OC. Second, nanocomposite based‐vaccines can coload OC antigens and immune adjuvants to further promote antigen presentation in OC immunotherapy (**Figure** **5**A).

### 4.1 Virus‐Like Nanoparticles (VLPs)

VLPs derived from plants and bacteria are highly immunogenic and activate pattern recognition receptors on many immune cells, such as macrophages, neutrophils, and DCs.^[^ ^78^ ^]^ VLPs can turn immune “cold” OC into “hot” OC and activate the immune system to fight OC.^[^ ^79^ ^]^ Among them, cowpea mosaic virus (CPMV), a 30 nm icosahedral nanoparticle consisting of a protein capsid,^[^ ^80^ ^]^ can effectively reverse the immunosuppressive TIM and stimulate local and systemic antitumor immune responses after CPMV in situ vaccination.^[^ ^81^ ^]^ Additionally, CPMV (as a plant virus) cannot replicate in mammalian cells and is safe as a cancer vaccine.^[^ ^82^ ^]^ In 2015, Lizotte et al. first adopted empty CPMV (eCPMV) as a novel nanovaccine for OC and found that eCPMV had an inherent immune stimulatory effect and induced infiltration of bone marrow‐derived dendritic cells (BMDCs) and M1 macrophages to secrete higher levels of typical proinflammatory cytokines (interleukin (IL)‐1*β*, IL‐6, IL‐12p40, MIP1‐*α*, and tumor necrosis factor (TNF)‐*α*) in OC mice. The eCPMV‐treated OC mice demonstrated a significantly increased survival rate and did not develop obvious ascites compared to phosphate‐buffered saline (PBS)‐injected controls.^[^ ^83^ ^]^

However, repeated injections of CPMV are required to maintain continuous immune stimulation for OC immunotherapy. Czapar et al. further developed a sustained‐release CPMV nanocarrier (CPMV–PAMAM) for OC immunotherapy.^[^ ^84^ ^]^ The CPMV–PAMAMs were prepared by coassembling negative CPMV with positively charged aminated 4‐polyamidoamine dendrimers (PAMAM). The high salt ion concentration of ascites shielded the charge in CPMV–PAMAM, which dissociated CPMV–PAMAM nanoparticles for the slow release of CPMV in ascites. As a result, the CPMV–PAMAMs were a CPMV storage to slowly release CPMV to prolong the immune stimulation time. A single intraperitoneal injection of CPMV–PAMAM triggered a sustained immune response to suppress OC growth and had the same effect as frequent administration of soluble CPMV in the mice model of abdominal OC.

Very recently, Wang et al. also developed an artificial active magnesium nanomotor (Motor) microcarrier (Q*β*‐motor) loaded with bacteriophage Q*β* (Q*β*) for OC immunotherapy.^[^ ^85^ ^]^ Q*β*‐motors were prepared by atomic deposition and layer‐by‐layer self‐assembly. The components of Q*β*‐motor from inside to outside were Mg core, TiO_2_ protective layer,^[^ ^86^ ^]^ and poly(lactic‐*co*‐glycolic acid) (PLGA) and Q*β* self‐assembly layer composed of chitosan (positive charge) and Q*β* (negative charge) (Figure 5B). Notably, the Q*β*‐motor had a small gap to expose the innermost Mg core, which allowed H^+^ to react with the Mg core to generate H_2_ bubbles to form a reverse driving force for the active movement of Q*β*‐motor in the slightly acidic environment of ascites. As a result, Q*β*‐motor actively delivered Q*β* into the deep part of the OC. After the treatment with Q*β*‐motor, the distribution and retention time of Q*β* were greatly improved in OC. In the OC model, free Q*β* was completely excreted 8 hours after the injection, while Q*β*‐motor extended the Q*β* retention time to 24 h. When Q*β*‐motor reached the tumor site, the secretion levels of IL‐6 and TNF‐*α* increased, while the level of IL‐10 decreased. Moreover, the survival time of mice was also extended from 70 days (free Q*β*) to 80 days after Q*β*‐motor treatment.

### 4.2 Nanocomposite‐Based Vaccines

VLPs must be administered to the site of OC lesions or efficiently enter the OC to increase antigen presentation of OC when VLPs are adopted alone as vaccines. However, this approach has great limitations, especially for tiny OC lesions or small OC metastases. Another effective solution is to coload immune adjuvants and OC antigens into nanomedicines, which can effectively promote the presentation of OC antigens to achieve cancer immunotherapy only by subcutaneous injection. For example, Patel et al. developed a nanocomposite‐based vaccine (CPMV‐NY‐ESO‐1) with CPMV as a nanocarrier to load cancer‐testis antigens (NY‐ESO‐1) to treat OC.^[^ ^87^ ^]^ NY‐ESO‐1 was highly expressed in OC cells and had been adopted as an OC‐specific antigen.^[^ ^88^ ^]^ In CPMV‐NY‐ESO‐1, CPMVs not only were used as an efficient immune adjuvant but also were modified with NY‐ESO‐1 through an amide bond to form CPMV‐NY‐ESO‐1. Compared with free NY‐ESO‐1, CPMV‐NY‐ESO‐1 significantly increased M1 macrophage uptake and level of inflammatory factors (e.g., TNF‐*α* by nearly twofold) to promote antigen presentation. Among them, TNF‐*α* effectively promoted the maturation and migration of DCs and increased antigen presentation of DC to T cells. After the subcutaneous injection of CPMV‐NY‐ESO‐1, CPMV‐NY‐ESO‐1 translocated to lymph nodes and ultimately promoted the production of NY‐ESO‐1‐specific CD8+ T cells, which were highly toxic to OC cells.

Additionally, VLPs can also be adopted in combination with ICIs to increase the therapeutic effect of immunotherapy.^[^ ^90^, ^91^ ^]^ For example, Gautam et al. covalently linked the PD‐1 antibody peptide (SNTSESF) to CPMV for OC immunotherapy.^[^ ^92^ ^]^ SNTSESF, also known as AUR‐7, is a potent ICI mimicking the endogenous PD‐1 receptor to inhibit PD‐1 function. CPMV–SNTSESF prepared by linking SNTSESF and CPMV through an amide bond. After CPMV–SNTSESF was inoculated in situ, CPMV increased the number of tumor antigen‐specific effector T cells, and SNTSESF blocked the combination of PD‐1 and PD‐L1 to prevent the immune escape of OC. The survival time of CPMV–SNTSESF‐treated mice has been significantly prolonged (75–99 days), and the treatment effect has been better than that of CPMV alone (71–75 days) and untreated mice (<57 days) in a mice model of metastatic OC.

Single or several OC antigen‐based vaccines easily lead to off‐target immunotherapy due to the heterogeneity of OC. This problem can be effectively avoided with autologous tumor cells (ATCs) as the source of OC antigens to prepare vaccines because ATCs contain various OC antigens. However, the instability of ATCs greatly limits their application as OC vaccines. Very recently, Guo et al. have developed a nanocomposite vaccine (Si‐PEI‐CpG‐MPL) for OC immunotherapy by enhancing ATC stability and antigen presentation.^[^ ^89^ ^]^ First, a nanoscale soluble silica shell was grown on OC cells by biomineralization and then has been cryogenically frozen to induce OC cell death to eliminate the possibility of tumorigenesis in vivo. To increase the presentation of OC antigens on ATC, a layer of polyethyleneimine (PEI) has been deposited on the silica surface, followed by loading with the immunoadjuvant cytosine–phosphate–guanine dideoxynucleotide motif (CpG) and monophosphoryl lipid A (MPL) (Figure 5C). CpG and MPL have been the agonists of toll‐like receptors (TLR) 9 and TLR 4 enriched on the surface of DCs, respectively, and effectively promoted the uptake and activation of Si‐PEI‐CpG‐MPL by DCs. After a subcutaneous or intraperitoneal injection, the proportion of effector memory T cells and TH1 polarization in CD4+ T cells was significantly increased, and the percentage of suppressive regulatory CD4+ T cells has been drastically reduced. Moreover, the levels of IL‐2, IFN‐*γ*, and TNF‐*α* produced by CD4+ and CD8+ T cells were also greatly increased. Si‐PEI‐CpG‐MPL significantly improved the survival rate of the BR5‐Akt tumor‐bearing mice (Figure 5D). The survival rate of OC mice with the Si‐PEI‐CpG‐MPL treatment group was as high as 100%, achieving complete tumor eradication and long‐term animal survival. Moreover, Si‐PEI‐CpG‐MPL had excellent stability and did not fail due to dehydration. Si‐PEI‐CpG‐MPL still had the same immune efficacy after being stored at room temperature for two weeks.

## 5 Nanomedicines Heat OC by Repolarizing TAM

**Figure 6:**
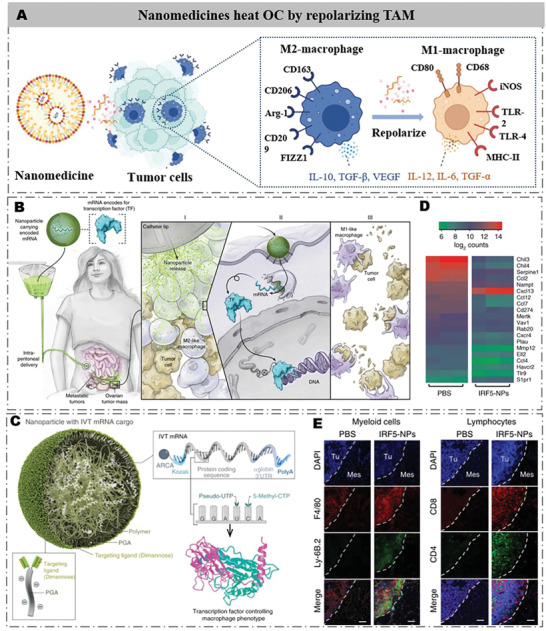
Development strategies for nanomedicine‐induced TAM repolarization. A) Schematic illustration of the nanomedicines heating OC by repolarizing TAM. Drug‐ or gene‐induced repolarization of tumor‐associated macrophages reverses the immunosuppressive TIM. B) An illustration of the planned clinical application, designed to treat OC patients with repeated intraperitoneal infusions of mRNA nanoparticles. C) Heat map of signature gene expression in macrophages isolated from mice treated with IRF5‐NPs versus control PBS. D) Design of macrophage‐targeted polymeric NPs formulated with mRNAs encoding key regulators of macrophage polarization. E) PBS or IRF5/IKK*β* NPs (50 µg mRNA/dose) have been injected and stained for the indicated myeloid and lymphocyte markers. Scale bar: 100 µm. Tu, tumor, Mes, mesentery. Reproduced with permission.^[^ ^101^ ^]^ Copyright 2019, Springer Nature.

Almost all TAMs are of the M2 type in OC, and TAMs can account for more than 50% of the total mass of solid tumors in advanced OC.^[^ ^93^ ^]^ Many TAMs originate from monocytes of the circulatory system by recruitment with cytokines, such as colony‐stimulating factor‐1, secreted by OC cells and then are converted to M2 macrophages at the tumor site.^[^ ^94^ ^]^ Given the abundance of TAM in the OC site, it is an effective and promising strategy to reshape the OC TIM (from cold to hot) by converting M2 type TAMs to M1 type at the OC site owing to the high flexibility of macrophages. Currently, many small molecule drugs, such as TLR agonists, cytokines, and CSF‐1R inhibitors, have been developed to promote TAM polarization.^[^ ^95^, ^96^ ^]^ However, these immunomodulators often have high systemic toxicity due to the lack of OC targeting and low bioavailability.^[^ ^97^ ^]^ Additionally, nucleic acid drugs can promote the conversion of macrophages from M2 to M1 by altering the expression of certain specific proteins. However, the instability of nucleic acid drugs in vivo limits their application. As mentioned above, macrophages can highly efficiently phagocytize large‐size nanoparticles.^[^ ^67^ ^]^ Therefore, tailored nanocarriers with these immunomodulatory drugs and nucleic acid drugs can efficiently reverse the cold TIM of OC by improving the targeting and stability of these drugs (**Figure** **6**A).

### 5.1 TAM Polarization by Immunomodulatory Nanomedicines

Recently, TLR agonist‐based nanomedicines have been developed and demonstrated excellent efficacy for reversing cold TIM of OC.^[^ ^98^, ^99^ ^]^ For instance, Kang et al. developed an anionic liposome‐loaded large‐size (≈400 nm) resiquimod (RSQ) to enhance OC targeting and reduce systemic toxicity of RSQ for OC immunotherapy.^[^ ^99^ ^]^ RSQ is a TLR agonist with the immunostimulatory ability to repolarize macrophages from M2 to M1 and induce Th1 responses through nuclear factor kappa‐B activation. The RSQ‐loaded liposomes efficiently accumulated in TAMs of OC with up to 80% uptake due to the positive charge on the cell membrane surface of TAMs^[^ ^67^ ^]^ and successfully repolarized TAMs to a proimmunogenic M1 state. After an intraperitoneal injection of RSQ‐loaded liposomes, the proportion of M1 and M2 was greatly increased in the OC tissue in OC mice. Furthermore, MHC class II and CD86 levels expression were elevated on DCs in the draining lymph nodes, and the CD8/Treg ratio was significantly increased in OC lesions, suggesting that heating of OC TIM has significantly increased tumor antigen presentation and promoted adaptive immunity in OC. Finally, RSQ‐loaded liposomes showed excellent therapeutic effect with complete tumor rejection (defined as no tumor signal over 250 days after tumor implantation) after coadministration with RSQ‐loaded liposomes and *α*PD1.

IL‐12 is another promising immunomodulator for remodeling cold TIM of OC because IL‐12 can repolarize M2‐type TAMs to M1‐type and enhance the cytotoxicity of CD8+ T cells and NK cells. However, the off‐target systemic toxicity of IL‐12 has limited its clinical translation for OC. To this end, Barberio et al. developed an efficient IL‐12‐based delivery system (poly‐l‐glutamate (PLE)‐IL‐12‐NP) to reverse the cold TIM of OC.^[^ ^100^ ^]^ PLE‐IL‐12‐NPs were prepared by layer‐by‐layer self‐assembly: IL‐12 with C‐terminal histidine first were linked to the liposome surface through the interaction of the histidine of IL‐12 and the nickel‐containing headgroup of the liposome surface. The polyelectrolyte bilayer was further adsorbed onto the liposomes of IL‐12 with the layer‐by‐layer self‐assembly. These alternately charged polymer layers acted as an effective barrier to decreasing the systemic exposure and toxicity of IL‐12. The IL‐12‐LbL‐NPs accurately attached to the surface membrane of OC cells for a long time because PLE at the terminal layer of IL‐12‐LbL‐NPs had specifically bonded to highly expressed glutamate receptors on the surface of OC cells. As a result, IL‐12‐LbL‐NPs were highly (>92%) selective for OC cell, and IL‐12 was efficiently released from IL‐12‐LbL‐NPs within 8–24 h. After an intratumoral injection, IL‐12‐LbL‐NPs effectively reversed cold TIM of OC, and M1 macrophage, DC, and CD8+ T cell populations were all significantly increased in the OC site. Therefore, IL‐12‐LbL‐NPs effectively inhibited the growth of OC and prolonged the survival of mice. Moreover, IL‐12‐LbL‐NP was not significantly toxic and did not cause weight loss compared with free IL‐12.

### 5.2 TAM Polarization by Nucleic Acid Drug Nanomedicines

Nucleic acid drug‐based nanomedicines have also been developed to reverse TAM of OC in recent years, mainly including in vitro‐transcribed (IVT) mRNA and microRNA (miR). Specially designed nanocarriers for nucleic acid drugs can greatly improve the stability and OC targeting of nucleic acid drugs in vivo. For example, Zhang et al. developed a dimannose coated poly(*β*‐aminoester) (PbAE) polymer nanocarriers to load IVT mRNA (IRF5/IKK*β* NPs) for TAM polarization in OC^[^ ^101^ ^]^ (Figure 6B). The IRF5/IKK*β* NPs consisted of two mRNAs: the first encodes interferon regulatory factor 5 (IRF5)‐induced TAM polarization toward the M1 phenotype, and the second encodes IKK*β*, a kind of kinase to phosphorylate and activate IRF5. The IRF5/IKK*β* NPs selectively targeted M2‐type TAMs through the specific binding of the macrophage mannose receptor 1 and dimannose (Figure 6C). The IRF5/IKK*β* NPs reduced the proportion of M2‐type TAMs from 43% ± standard error (SE)/15.6–2.6% ± SE/2.1% and increased the proportion of M1‐type macrophages from 0.5% ± SE/0.2–10.2% ± SE/4.1% in OC mice (Figure 6D). Moreover, the IRF5/IKK*β* NPs increased neutrophil density by 16.2‐fold and T cell infiltration by an average of 10.6‐fold (CD8+) and 3.5‐fold (CD4+) in OC. Additionally, significant tumor regression was observed in the IRF5/IKK*β* NPs‐treated group compared with the control group in a mouse model of unresectable advanced OC (Figure 6E).

Similarly, Parayath et al. developed hyaluronic acid (HA)‐based nanoparticles as a delivery system for miR‐125b (HA‐PEI‐miR‐125b) to induce phenotypic transformation of TAM in OC.^[^ ^102^ ^]^ Cationic PEI were modified at the HA backbone to facilitate the encapsulation of miR‐125b to prepare HA‐PEI‐miR‐125b. miR‐125b efficiently repolarized M2 TAM to M1, and HA of HA‐PEI‐miR‐125b targeted the highly expressed integral membrane glycoprotein CD44 receptor on TAM. After HA‐PEI‐miR‐125b intraperitoneal administration, the number of CD206+ M2 macrophages decreased, that of antitumor CD80+ M1 macrophages increased by >2 times, and the ratio of iNOS (M1 marker)/Arg‐1 (M2 marker) increased by >10‐fold in a homologous ID8 OC mouse model.

## 6 Nanomedicines Improve OC Antigen Presentation

**Figure 7:**
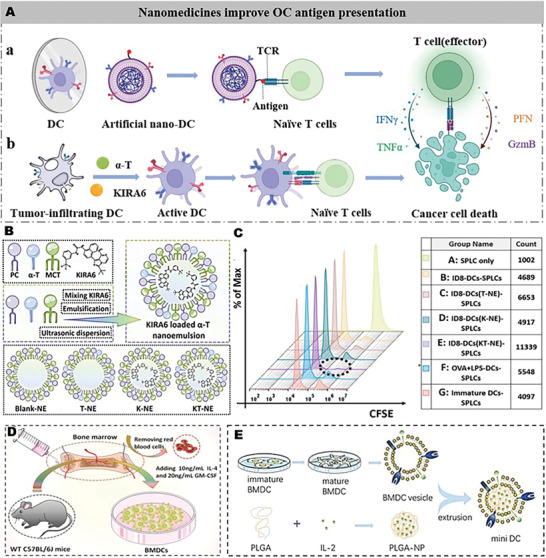
Development strategies for nanomedicines to restore or mimic DC function to increase antigen presentation. A) Schematic illustration of nanomedicines improving OC antigen presentation. (a) Restoring DC function or (b) developing artificial DCs to enhance antigen presentation and activate T cells. B) Schematic illustration of the preparation process of blank‐nanoemulsion (Blank‐NE), *α*‐tocopherol‐nanoemulsion (T‐NE), KIRA6‐nanoemulsion (K‐NE), and KT‐NE. C) The proliferation of SPLCs primed by DCs or PBS‐, T‐NE‐, K‐NE‐, and KT‐NE‐treated TIDCs were evaluated by CFSE dilution. D) Cellular morphology of murine BMDCs generated in vitro. Reproduced with permission.^[^ ^112^ ^]^ Copyright 2022, Elsevier Ltd. E) Schematic illustration for the preparation of a mini DC. Reproduced under the terms of a CC‐BY license.^[^ ^61^ ^]^ Copyright 2020, The Authors. Published by Wiley‐VCH.

Cancer‐specific immunity begins with antigen presentation by DCs.^[^ ^103^ ^]^ However, the number of DCs is very low in the TIM of OC, and tumor‐infiltrating DCs (TIDCs) located near the OC (tumor bed or draining lymph nodes) are severely hampered in terms of immune activity.^[^ ^103^ ^]^ Abnormal proliferation of OC cells leads to high metabolic demand and insufficient O_2_ supply to trigger oxidative stress by producing excessive reactive oxygen species (ROS) in TME.^[^ ^104^, ^105^, ^106^ ^]^ As a result, intracellular concentrations of reactive aldehyde 4‐hydroxynonenal (4‐HNE), a byproduct of lipid peroxidation, are elevated in TIDCs.^[^ ^107^, ^108^ ^]^ 4‐HNE induces endoplasmic reticulum (ER) stress to lead to hyperactivation of inositol‐requiring kinase 1*α* (IRE1*α*)‐X‐box binding protein 1 (XBP1).^[^ ^109^, ^110^ ^]^ XBP1 promotes aberrant lipid accumulation to manipulate metabolic signaling for DCs deactivation by enhancing fatty acid oxidation, which is closely related to the immature resting phenotype of DCs with the little secretion of cytokines and weak ability to activate T cells.^[^ ^111^ ^]^ Collectively, lipid accumulation and ER stress inhibit TIDC activation in OC. Currently, two classes of nanomedicines are developed to overcome these problems to increase antigen presentation of DCs: the first is to restore the function of DCs with nanomedicines, and the second is an artificial nano‐DC with biomimetic nanomaterial to promote OC antigen presentation (**Figure** **7**A).

Very recently, Lu et al. developed a nanoemulsion (KT‐NE) loaded with KIRA6 and *α*‐tocopherol for restoring DC function to reverse inhibitory TIM in OC^[^ ^112^ ^]^ (Figure 7B). *α*‐tocopherol effectively relieved oxidative stress and decreased 4‐HNE production as a powerful ROS scavenger. Correspondingly, KIRA6, an advanced small‐molecule kinase and RNase inhibitor of IRE1*α*, reduced IRE1*α*‐oligomerization to induce cleavage of XBP1 mRNA and inhibit XBP1‐mediated synthesis of fatty acids. As a result, KT‐NE coinhibited XBP1 hyperactivation and ROS‐triggered lipid peroxidation to ameliorate dysfunctional TIDC. Strikingly, KT‐NE‐treated ID8‐DCs significantly promoted T cell expansion, with nearly 97% of syngeneic splenic lymphocytes (SPLCs) proliferation within 24 h. Meanwhile, SPLCs (effector T cells) activated by ID8‐DCs (KT‐NE) exhibited excellent antitumor efficacy, with ≈90% of ID8 tumor cells being lysed (Figure 7C,D). Furthermore, the coadministration of KT‐NE with *α*PD‐1 significantly delayed the progression of aggressive high‐grade serous OC and almost eliminated intraperitoneal metastases in the abdominal cavity.

Artificial biomimetic nano‐DCs are also an effective method to increase antigen presentation of OC and have higher stability and controllability than natural DCs.^[^ ^61^, ^113^ ^]^ For example, Cheng et al. developed DC‐like nanoparticles (mini DCs) to increase antigen presentation of OC.^[^ ^61^ ^]^ Mini DCs were prepared by a two‐step process. First, biodegradable PLGA nanoparticles were efficiently loaded with IL‐2. Then, their surface was coated with a layer of DC membrane (Figure 7E). The coating cell membrane of mini DCs inherited all DC functional properties of membrane proteins, such as MHC, CD86, and CD40, and mimicked the antigen presentation ability of DCs. Additionally, IL‐2 were slowly released to activate T cells and stimulate a powerful and continuous antitumor immune response when PLGA of mini DCs were gradually degraded in vivo. Therefore, mini DCs was not affected by physiological regulation in the process of antigen presentation and immunosuppressive TIM of OC. Moreover, mini DCs had feasible storage conditions and longer shelf life to provide better clinical operability compared with DCs. In the OC‐bearing mice model, mini DCs stimulated the production abundant of CD3+CD8+ T cells and reduced CD4+ CD25+ Foxp3+ regulatory T cell (Treg) levels to inhibit tumor growth after mini DCs administration.

## 7 Nanomedicines Increase T Cell Infiltration

**Figure 8:**
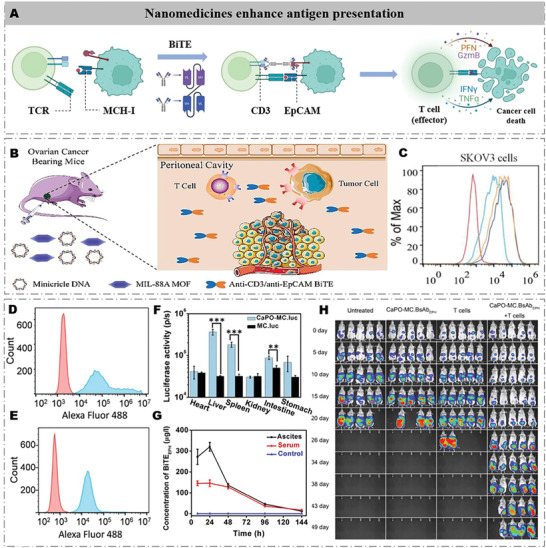
Development strategies for nanomedicines to increase T cell homing properties. A) Schematic illustration of nanomedicines increasing T cell infiltration. Bispecific T receptors recruit T cells to infiltrate tumors and promote T cell binding to cancer cells. B) Schematic diagram of MC‐MIL‐88A development strategy. C) Binding specificity of anti‐CD3/anti‐EpCAM to EpCAM‐positive SKOV3 cells at different concentrations of BiTE. Reproduced with permission.^[^ ^117^ ^]^ Copyright 2020, Elsevier Ltd. D) Binding of BsAb_EPH_ on CD3+ T cells. E) Binding of BsAb_EPH_ on EpCAM‐positive SKOV3 cells. F) A chart comparing luciferase activity in individual organs from the mice 8 h post‐transfection with MC encoding luciferase reporter gene (MC.luc). G) BsAb_EPH_ expression in mice after intraperitoneal injection of CaPO‐MC.BsAb_EPH_ complex. H) Bioluminescence images of individual mice. Reproduced with permission.^[^ ^121^ ^]^ Copyright 2020, Elsevier Ltd.

T cell killing is the last and direct part of immunotherapy, but this process is severely hindered by the lack of T cell infiltration in OC. Therefore, an effective approach is to recruit T cells to the TIM and facilitate their binding to tumor cells. BiTEs are a class of artificial antibodies with two antigen‐binding sites, which can specifically bind to T cell surface antigen and cancer cell surface antigen. Therefore, BiTEs are a class of promising drugs to increase T cell infiltration (**Figure** **8**A). Currently, catumaxomab was certified by the European Union for the treatment of OC in 2009.^[^ ^114^ ^]^ The two single‐chain fragments of catumaxomab are T cell‐specific monoclonal antibody (anti‐CD3) and OC cell surface antigen monoclonal antibody (anti‐EpCAM), which can promote the connection between T and OC cells to activate T cells to kill OC. However, catumaxomab has a short half‐life in vivo and requires continuous administration to reach an effective dose.^[^ ^115^, ^116^ ^]^

Nanomedicines can effectively solve this bottleneck by directly expressing BiTEs at the OC site. For instance, Jing et al. developed an MIL‐88A‐based catumaxomab coding gene nanocarriers (minicircle DNA (MC)‐MIL‐88A) for OC immunotherapy^[^ ^117^ ^]^ (Figure 8B). MC was adopted as a nonviral gene vector for catumaxomab due to many advantages, including small size, high transfection efficiency, and high biological safety. MIL‐88A was used as a safe and efficient MC carrier because MIL‐88A efficiently bonded DNA by Fe^3+^‐phosphate coordination and electrostatic interactions.^[^ ^118^, ^119^, ^120^ ^]^ After an intraperitoneal injection of MC‐MIL‐88A, catumaxomab highly and consistently expressed with the peak concentration of 77.3 ng mL^−1^ (serum) and 51.0 ng mL^−1^ (ascites) in the OC xenograft model (Figure 8C). At OC sites, MC‐MIL‐88A effectively increased CD3+ T cell infiltration, and the median survival time of model mice was extended from 26 to 39 days. Very recently, Zhao et al. developed a needle‐like calcium phosphate nanocarrier with higher transfection efficiency for catumaxomab‐coding MC (CaPO‐MC) to treat OC.^[^ ^121^ ^]^ The needle‐shaped calcium phosphate nanocarriers effectively pierced the cell membrane to transfect catumaxomab‐coding MC into cells (Figure 8D,E). The maximum amounts of catumaxomab expressed by CaPO‐MC reached 146.14 ng mL^−1^ (serum) and 320.37 ng mL^−1^ (ascites) (Figure 8F,G), and the survival period of OC model mice was extended from 22 days to 40–58 days (Figure 8H).

## 8 Multifunctional Nanomedicines Heat Tumors Through ICD

**Figure 9:**
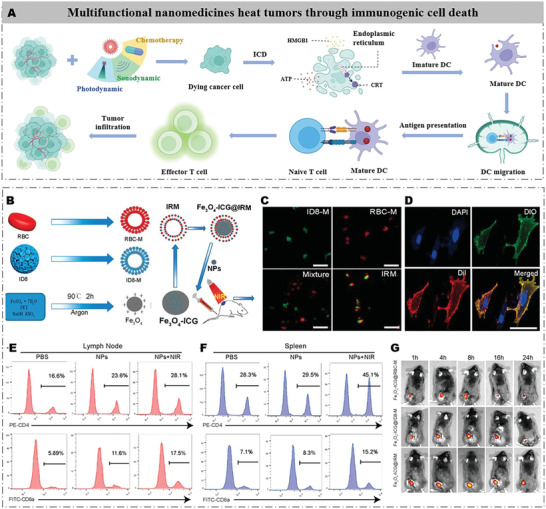
Development strategy of nanomedicine‐induced ICD for multitherapeutic OC immunotherapy. A) Schematic illustration of multifunctional nanomedicines heating tumors through ICD. Photodynamic/sonodynamic therapy induces immunogenic death of dying cells to trigger the immune response. B) Preparation processes of Fe_3_O_4_‐ICG@IRM nanoparticles. C) CLSM images of ID8‐M, RBC‐M, a mixture of ID8‐M and RBC‐M, and the fused IRM vesicles. Scale bars = 5 µm. D) CLSM images of fused IRMs incubated with ID8 cells for 3 h. Scale bars = 20 µm. E) The proportion of CD4+ and F) CD8a+ cells in the tumor‐draining lymph nodes and the spleen at 48 h after intratumoral injection of Fe_3_O_4_‐ICG@IRM in the left flank of ID8 bilateral tumor‐bearing mice with or without NIR radiation. G) In vivo fluorescence images showing tumor retention of Fe_3_O_4_‐ICG@RBC‐M, Fe_3_O_4_‐ICG@ID8‐M, and Fe_3_O_4_‐ICG@IRM over 24 h. Reproduced with permission.^[^ ^127^ ^]^ Copyright 2021, American Chemical Society.

Nanomedicines can also serve as a multifunctional platform to enhance the efficacy of immunotherapy owing to their high component selectivity and functional modification. Chemotherapy and other many emerging cancer treatments, such as photothermal therapy (PTT),^[^ ^122^ ^]^ photodynamic therapy,^[^ ^123^ ^]^ and sonodynamic therapy,^[^ ^124^, ^125^ ^]^ have been recently found to greatly facilitate the transition of the OC from cold to hot through ICD. Under these treatments, dying OC cells release abundant damage‐associated molecular patterns (DAMPs), such as calreticulin, adenosine‐5′‐triphosphate (ATP), high mobility group box 1 (HMGB1), etc. Among them, ATP can trigger the rapid infiltration of macrophages and DCs in OC. HMGB1, as a proinflammatory signal, can bind with TLR4 on macrophages and DCs to promote inflammatory cell infiltration and antigen presentation. Calreticulin also promotes phagocytosis of inflammatory cells after ectopic from the endoplasmic reticulum to the cell membrane.^[^ ^126^ ^]^ Therefore, ICD induced by these therapies can greatly reverse the cold OC and promote the effect of immunotherapy (**Figure** **9**A).

For example, Xiong et al. reported a multifunctional nanomedicine (Fe_3_O_4_‐ICG@IRM) for the treatment of OC with enhanced immunotherapy by PTT‐induced ICD.^[^ ^127^ ^]^ Fe_3_O_4_‐ICG@IRM were prepared by coating a hybrid membrane (IRM) of erythrocytes and OC cells on indocyanine green (IGG)‐loaded Fe_3_O_4_ nanoparticles (Figure 9B). In Fe_3_O_4_‐ICG@IRM, Fe_3_O_4_ and ICG were used as PTT reagents, and the two combinations synergistically increased the photothermal efficiency. Additionally, the coating of IRM effectively increased the lifespan of Fe_3_O_4_‐ICG@IRM in the blood and the tumor site‐specific accumulation with homologous targeting to OC cell because IRM had the same properties as erythrocyte membrane and OC membrane (Figure 9C,D). After irradiation with near‐infrared light (808 nm), Fe_3_O_4_‐ICG@IRM significantly induced ID‐8 cell death and released abundant DAMPs to activate ICD, which promoted M1 macrophage infiltration and produced a high level of inflammatory factors (TNF‐*α*, IL‐6, and IL‐1*β*). Moreover, PTT further induced adaptive immunity in OC. The number of CD4+ and CD8+ T cells was significantly increased in the OC site, and the mean percentages of CD80+ CD86+ DCs and CD80+ F4/80+ macrophages were increased from 10.1% to 18.4% and 7.5% to 15.1%, respectively, in lymph nodes (Figure 9E,F). Therefore, adaptive immunity based on this multifunctional nanoplatform had an excellent antitumor effect on metastatic OC (Figure 9G). Immune adjuvants and immune checkpoint inhibitors can further increase the effect of PTT‐based ICD for immunotherapy. Recently, Cao et al. developed a copper sulfide‐based nanoplatform loaded with CpG and *α*PD‐1 (CuS NPs/*α*PD‐1/CpG) to coadminister PTT and immunotherapy for OC.^[^ ^122^ ^]^ Copper sulfide nanoparticles are a class of efficient photothermal reagents and induce ICD of OC cells under near‐infrared region II laser irradiation (1064 nm).^[^ ^128^ ^]^ The combined administration effect of CuS NPs/*α*PD‐1/CpG was significantly better for the survival time of mice than that of *α*PD‐1 administration alone, owing to the synergistic effect of PTT, TLR agonists (CpG), and ICI (*α*PD‐1).

Digoxigenin (Dig) can further increase the ICD of multifunctional nanomedicines to improve the efficacy of OC immunotherapy. Xiang et al. recently reported a multidrug‐loading nanosystem (NCP) with Dig to enhance OC immunotherapy by chemotherapy‐based ICD.^[^ ^129^ ^]^ NCP was prepared by a reverse microemulsion method with the coordination of Zn^2+^ with 1,2‐dioleoyl‐sn‐glycero‐3‐phosphate. CbP/siPD‐L1@Dig NCPs were loaded with three kinds of drugs: carboplatin‐bis (phosphoramidic acid) (CbP, as a chemotherapeutic drug), siPD‐L1 (reducing PD‐L1 expression, as an ICI), and Dig. The CbP/siPD‐L1@Dig NCP was rapidly ruptured in acidic organelles after endocytosis, simultaneously initiated a caspase cascade via Carb, and overcome immunosuppression via PD‐L1 silencing. Notably, Dig, as an ICD enhancing drug,^[^ ^130^ ^]^ significantly enhanced chemotherapy‐induced ICD.^[^ ^127^ ^]^ CbP/siPD‐L1@Dig‐NCP‐ and CbP/siPD‐L1‐NCP‐treated OC cells exhibited up to 22‐fold and 35‐fold increase in the concentration of the ICD‐related marker ATP, respectively, compared to untreated OC cells. The CbP/siPD‐L1@Dig NCP group shown significant tumor‐suppressive and antimetastatic effects with a median survival of 44 days in a mouse model of invasive metastatic OC with widespread intraperitoneal dissemination.

## 9 Summary and Prospects

Immunotherapy has significantly improved the survival of advanced melanoma, lung cancer, and other cancers and triggered a revolutionary change in cancer treatment.^[^ ^131^, ^132^ ^]^ Many other types of cancer have also been extensively explored with immunotherapy to improve survival, but with only limited success. For example, ICIs provide significant improvement in only about 13% of overall cancer patients.^[^ ^133^ ^]^ The cold tumor microenvironment is the main reason for immunotherapy failure.^[^ ^134^ ^]^ However, many approaches aimed at remodeling the TIM are often associated with severe systemic toxicity, frequent dosing, and only modest clinical efficacy.^[^ ^135^ ^]^ Nanomedicines are revealed as effective tools to overcome the limitations of these cold TIM.^[^ ^136^ ^]^ In this review, we took a typical cold tumor‐OC as a paradigm to introduce five general nanomedicine‐based strategies to greatly improve the effectiveness of immunotherapy by heating the TIM of OC. All the above‐mentioned strategies are developing into exciting research fields of OC therapy. Overall, this review provided timely inspiration and reference for the design of nanomedicines for the remodeling of the TIM of OC or many other cold tumors (**Table** **2**). Despite the great progress in preclinical research, there are some limitations that urgently need to be considered. In this section, we list some challenges and suggest potential future directions.

### 9.1 Obstacles to the Clinical Translation of Nanomedicines

Hundreds of thousands of scientific papers on nanomedicine have been published, but only a few studies have reached clinical trials for various applications. This huge gap has sparked doubts and even disbelief among researchers in nanomedicines. However, there is a widely overlooked fact that the estimated overall success rate of cancer nanomedicines from phase I to clinical approval is 6%, much higher than the 3.4% for conventional new medicines.^[^ ^137^ ^]^

However, it is undeniable that the clinical translation of nanomedicine is facing some obstacles that need to be solved. First, the balance between the complexity of nanomedicine components, manufacturing cost, and immunotherapy effect should be considered to realize the clinical translation of nanomedicines.^[^ ^138^ ^]^ It is also necessary to take into account the huge impact of the complexity of the nanomedicine manufacturing process on its efficacy, ensuring the consistency and stability of different batches of nanomedicines is necessary to obtain long‐term benefits. Another obstacle to the clinical translation of nanomedicines is the unacceptable mismatch between laboratory research results and clinical application effects. Many events that go unnoticed, inadvertent, or unobservable at the laboratory level suddenly manifest clinically and lead to translational failure.^[^ ^138^ ^]^ Thus, the biocompatibility, toxicity, and side effects of nanomedicines should be further studied. For example, the toxicity of these preclinical nanomedicines is generally only studied for a month or a few months, which is far from enough. Therefore, the long‐term toxicity of nanomedicines, including chronic inflammatory and immune responses caused by their degraded metabolites, and other side effects need to be studied in depth.^[^ ^139^ ^]^ In addition, it may be questioned that pharmacokinetic information such as absorption and metabolism of nanomedicines is unknown or uncontrollable. However, these problems will be solved as long as sufficient and reliable trails have been done before a nanomedicine entering the clinic. On the other hand, the FDA currently does not have a complete regulatory system for nanomedicines. This is because any single “nanomedicine” may contain many different biologically active compounds, each with its own properties, and create a completely different compound with new properties. The FDA requires that every ingredient added to a nanomedicine needs its own justification and testing, especially if those ingredients are previously unapproved compounds.^[^ ^140^ ^]^ The versatility of nanomedicines maximizes regulatory barriers to nanomedicine translation.

Despite the above obstacles, there are also many nanomedicines that have played a substantial role in the clinic. Take liposome as an example, which has achieved obvious success in clinical applications over the past 40 years. The annual sales of AmBisome and Doxil both reach hundreds of millions of dollars.^[^ ^141^, ^142^ ^]^ In addition, iron oxide nanoparticles (Feraheme), albumin nanoparticles (Abraxane) and other materials are also widely used in clinical practice.^[^ ^143^, ^144^ ^]^ Therefore, we remain optimistic about the potential of nanomedicines in cancer treatment.

### 9.2 Differences between Animal Models and OC Patients

For cancer treatment, the attractive efficacy of preclinical studies is not always confirmed in clinical trials. One important reason is that the models of preclinical studies are different from OC patients.^[^ ^145^ ^]^ Current experiments with these nanomedicines are performed in small animal models, which are usually limited to replicating one or a few aspects of the OC TIM rather than the overall complexity. For example, these animal models rarely involve the complex and abnormal vasculature of OC, which has great implications for immunotherapy. Emerging organoid cultures may offer an effective solution to this problem by simulating the real OC microenvironment and aid with further screening and optimization of nanomedicines.^[^ ^146^ ^]^ Furthermore, the OC TIM is often closely related to estrogen levels and microbes in the environment. Innate and adaptive immunity is significantly upregulated in model animals when laboratory model animals are housed with pets.^[^ ^147^ ^]^ Additionally, many OC patients are postmenopausal or have undergone OC resection.^[^ ^148^ ^]^ Estrogen levels can significantly affect immune cell function to interfere with the immunotherapy. Therefore, an effective solution is to simulate the real situation of OC patients, such as adopting small animals exposed to unscreened microbial environments or ovariectomized small animals as models.

### 9.3 Stratification of OC Patients

Facilitation of the clinical translation of nanomedicines is also extremely important for OC patients’ stratification.^[^ ^149^ ^]^ Many nanomedicines with excellent efficacy in preclinical research have efficacy only in some patients due to the heterogeneity of OC.^[^ ^150^ ^]^ For example, not all OC patients have significant EPR effects. After stratifying patients with appropriate diagnostic techniques, OC patients with EPR can be effectively treated by nanomedicines, and patients with less EPR can avoid the drug resistance caused by nanomedicine treatment. Fortunately, recent nanoprobes can quantify EPR effects by magnetic resonance imaging or positron emission tomography‐computed tomography.^[^ ^151^, ^152^ ^]^ Therefore, nanomedicine‐sensitive OC patients can benefit from immunotherapy and promote the clinical translation of nanomedicines by stratifying heterogeneous OC patients.

### 9.4 Reversing the TIM Through a Nonimmune Cellular Pathway

To heat OCs, current nanomedicines target almost all immune cells or OC cells. However, targeting immune cells may affect systemic immunity, which is critical for patient survival. In fact, cold OC TIM is also closely related to abnormal vasculature, fibroblasts, extracellular matrix,^[^ ^153^ ^]^ hypoxia, and a slightly acidic environment.^[^ ^154^, ^155^ ^]^ The immune microenvironment can also be effectively reversed by altering noninflammatory cytokines without affecting systemic immunity. For example, the hypoxic environment of tumors can greatly promote the expression of hypoxia‐inducible factor 1*α* (HIF‐1*α*). HIF‐1*α*, as an important nuclear transcription factor, can promote the expression of immune checkpoint ligands, such as PDL‐1, CD80, and CD86, and lead to immunosuppression at tumor sites.^[^ ^156^ ^]^ As a result, the effect of nanomedicines can be further increased to promote immunotherapy by normalizing these factors as the next step in research.

The ultimate immunotherapy success of many cold cancers depends on the degree to shift its cold TIM to hot.^[^ ^157^, ^158^ ^]^ From these preclinical studies, we can see the dazzling effect and great prospect of nanomedicines in reversing cold OC TIM. In response to the above‐mentioned challenges, the next generation of nanomedicines is expected to achieve clinical applications and ultimately improve the health of OC patients with the joint efforts of scientists from various disciplines, such as materials science, nanoscience, chemistry, biology, medicine, and pharmacy.

**Table 2:** Nanomaterials in immunotherapy of OC

Categories	Material	Contents	Administration	Curative effect	Refs.
Nanovaccines “heating” OC	CPMV	Tumor cell lysates	Intraperitoneal injection	Increased T cells infiltration	[91]
	CPMV	NY‐ESO‐1	Subcutaneous injection	Increased CD8+T cells, TNF‐*α*, IL‐1*β*, IL‐6, and IL‐12p70	[87]
	CPMV	SNTSESF	Intraperitoneal injection	Increased T cells infiltration	[92]
	CPMV	CD47 blocker	Intraperitoneal injection	Increased MI TAMs, IL‐6, and TNF‐*α*	[90]
	CPMV G4‐PAMAM	–	Intraperitoneal injection	Prolonged CPMV duration of action	[84]
	CPMV	–	Intratumoral injection	Increased CD8/4+T cells infiltration	[159]
	eCPMV	–	Intratumoral injection	Increased activated T cells, N1 TANs, DCs NK cells, and CXCL9/10	[83]
	Magnesium nanomotor	Bacteriophage Q*β*	Intraperitoneal injection	Increased antitumor TAM and TAN, IL‐6, and TNF‐*α*; Reduced IL‐10	[85]
	Silica NPs	ATC	Subcutaneous or intraperitoneal injection	Increased CD8/4+ T cells, IL‐2, IFN‐*γ*, and TNF‐*α*	[89]
Nanomedicines heat OC by repolarizing TAM	Liposomes	RSQ	Intraperitoneal injection	Increased M1 TAMs and CD8+T cells; Reduced M2 TAMs and Treg	[67, 99]
	Very small size particles	–	Intraperitoneal injection	Increased the proportion of M1 and M2 TAMs	[98]
	Liposomes	IL‐12	Subcutaneous injection	Increased populations of CD8+ T cells, DCs, neutrophils, and macrophages	[100]
	HA‐PEI	miR‐125b	Intraperitoneal injection	Increased the proportion of M1 and M2 TAMs more than 2 times	[102]
	PbAE	IVT mRNA (IRF5/IKK*β*)	Intraperitoneal injection	Increased CD8/4+ T cells, neutrophils, and the proportion of M1 and M2 TAMs	[101]
Nanomedicines improve OC antigen presentation	Nanoemulsion	*α*‐Tocopherol KIRA6	Subcutaneous injection	Increased activated DCs and T cells; Reduced lipid accumulation in TIDCs	[112]
	PLGA	IL‐2 DC membrane	Subcutaneous injection	Increased effective T cell infiltration and secretion of IFN‐*γ* and TNF‐*α*; Reduced Treg	[61]
	PLGA	DC membrane ID8 membrane CpG‐ODN	Subcutaneous injection	Increased CD8+T cell and CTL; Reduced Treg	[113]
Nanomedicines increase T cell infiltration	CaPO Nanoneedle	Catu‐maxomab BiTEs	Intraperitoneal injection	Increased T cell, IFN‐*γ*, and TNF‐*α*	[121]
	MIL‐88A metal‐organic framework	Catu‐maxomab BiTEs	Intraperitoneal injection	Increased T cell, TNF‐*α*, TFN‐*γ*, and granzyme B	[117]
Multifunctional nanomedicines heat tumors through ICD	DSPE‐PEG2K	siPD‐L1, Cb, Dig	Intravenous injection	Increased CLTs and M1 TAMs; Reduced Treg, M2 TAMs, and MDSCs	[129]
	CuS NPs	*α*PD‐1, CpG	Intravenous injection	Increased CD8+T cell and DC	[122]
	Fe_3_O_4_ NPs	RBC membrane ID8 membrane ICG	Intravenous injection	Increased CD8+T cell and secretion of TNF‐*α*, TFN‐*γ*, and granzyme; Reduced Treg	[127]
	PLGA	PFP, ICG, OXP	Subcutaneous injection	Increased ICD and CLTs	[124, 160]
	PLG‐g‐mPEG	RSQ cisplatin	Intravenous injection	Increased M1 TAMs and apoptosis‐related gene expression	[161]

## Conflict of Interest

The authors declare no conflict of interest.

## Authors Contribution

Y.Y. and T.Z. contributed equally to this work. All authors have made a substantial, direct, and intellectual contribution to the work.
